# A prospective, multi-center, phase IV clinical study to assess the safety and clinical outcome of acalabrutinib in Indian adult patients with chronic lymphocytic leukemia/small lymphocytic lymphoma or relapsed/refractory mantle cell lymphoma

**DOI:** 10.3389/fonc.2026.1822178

**Published:** 2026-09-10

**Authors:** Pankaj Malhotra, Dinesh Bhurani, Manju Sengar, Nitin Sood, Arijit Nag, Anil Aribandi, Biju George, Hari Menon, Jina Bhattacharyya, Mallikarjun Kalashetty, Narendra Agrawal, Neeraj Sidharthan, Rayaz Ahmed, Sandip Shah, Saurav Bhave, Sharat Damodar

**Affiliations:** 1 Postgraduate Institute of Medical Education and Research, Chandigarh, India; 2 Rajiv Gandhi Cancer Institute & Research Centre, Delhi, India; 3 Tata Memorial Hospital, Mumbai, Maharashtra, India; 4 Medanta, The Medicity, Gurugram, Haryana, India; 5 Tata Medical Center, Kolkata, West Bengal, India; 6 American Oncology Institute, Hyderabad, Telangana, India; 7 Christian Medical College, Vellore, Tamil Nadu, India; 8 Cytecare Cancer Hospitals Pvt. Ltd, Bengaluru, Karnataka, India; 9 Guwahati Medical College and Hospital, Guwahati, Assam, India; 10 Manipal Hospital, Bengaluru, Karnataka, India; 11 Amrita Institute of Medical Sciences, Kochi, Kerala, India; 12 Hemato Oncology Clinic, Ahmedabad, Gujarat, India; 13 Vedant Hospital, Thane/Mumbai, Maharashtra, India; 14 Mazumdar Shaw Medical Centre, Narayana Hrudayalaya Limited, Bengaluru, Karnataka, India

**Keywords:** acalabrutinib, adverse events, Bruton tyrosine kinase inhibitors, chronic lymphocytic leukemia/small lymphocytic lymphoma, post-marketing study, real-world setting, safety profile

## Abstract

**Objective:**

To evaluate the safety and clinical outcomes of acalabrutinib in patients with chronic lymphocytic leukemia/small lymphocytic lymphoma (CLL/SLL) or refractory/relapsed mantle cell lymphoma (MCL) in a real-world setting in India.

**Methods:**

This Phase IV, open-label, single-arm, multi-center, prospective study included 89 patients with CLL/SLL (treatment naive or one or more prior therapy) and 11 patients with refractory/relapsed MCL who received acalabrutinib capsules 100 mg orally twice daily for six 28-day cycles or until study drug discontinuation. Patients were followed up for 28 days after the drug discontinuation to collect additional safety data. The primary endpoint was the incidence of adverse events (AEs), treatment-emergent AEs (TEAEs), serious adverse events (SAEs), second primary malignancies, and other AEs of clinical interest. Secondary endpoints included objective response rate (ORR) and quality of life.

**Results:**

By the end of the follow-up period, 48 (54%) patients in the CLL/SLL cohort and 9 (82%) patients in the MCL cohort reported at least one AE. Of these, 38 (43%) patients in the CLL/SLL cohort and 7 (64%) in the MCL cohort reported TEAEs, with serious TEAEs reported in 9% and 27%, respectively. Three patients in CLL/SLL and two in MCL reported drug-related TEAEs. Drug-related deaths were reported in two MCL patients. Drug discontinuation due to AEs was reported in 3.4% and 18.2% of the CLL/SLL and MCL cohorts, respectively. During the follow-up period, the ORR was 62% in CLL/SLL cohort and 36% in MCL cohort, with quality of life scores maintained throughout.

**Conclusion:**

The study demonstrated safety of Acalabrutinib with no new signals and encouraging clinical outcomes in Indian patients with CLL/SLL and MCL, consistent with global clinical trials.

**ClinicalTrials.gov identifier:**

NCT04930536.

## 1 Introduction

Chronic lymphocytic leukemia/small lymphocytic lymphoma (CLL/SLL) is a relatively rare hematological malignancy in Asia, with patients typically presenting at a younger age compared with Western countries (1–4). Treatment options for CLL/SLL range from observation in asymptomatic patients (3, 4) to targeted therapy for patients with active disease. While chemotherapy, chemo-immunotherapy (5), and hematopoietic stem cell transplantation (6) were historically the mainstay of treatment, contemporary management has largely shifted toward targeted therapies, including Bruton tyrosine kinase (BTK) inhibitors (such as acalabrutinib, zanubrutinib, and pirtobrutinib) and B-cell lymphoma 2 (BCL2) inhibitors (such as venetoclax) with or without anti-cluster of differentiation 20 (CD20) monoclonal antibodies (such as obinutuzumab or rituximab) (7). Targeted therapies offer improved disease control and reduced toxicity compared with conventional chemotherapy-based approaches (7). Nevertheless, the substantial healthcare cost poses challenges to their widespread accessibility in resource-constrained settings such as India (8, 9).

BTK inhibitors have transformed the treatment landscape of CLL/SLL, by targeting the B-cell receptor signaling pathway, which is critical for tumor cell survival (10, 11). The first-generation BTK inhibitor, ibrutinib, showed superior efficacy and an improved safety profile compared with conventional chemo-immunotherapy in both treatment-naïve and relapsed/refractory patients, including those harboring high-risk genetic markers such as del(17p) or TP53 mutations (10, 11). Consequently, ibrutinib is widely regarded as a standard of care for the treatment of CLL/SLL (12, 13). However, long-term use of ibrutinib is associated with adverse events, particularly cardiovascular toxicities, including atrial fibrillation and hypertension (14, 15). This led to the development of next-generation BTK inhibitors with improved selectivity and tolerability, predominantly shifting the therapeutic approach for CLL/SLL away from chemo-immunotherapy (16–18).

Acalabrutinib, a second-generation BTK inhibitor, has shown comparable effectiveness to ibrutinib, with an improved safety profile and less cardiovascular toxicity in clinical studies (19). This can be attributed to an increased selectivity of acalabrutinib as a BTK inhibitor, resulting in less off-target effects and higher *in vivo* potency (20, 21). In the ELEVATE-RR study, acalabrutinib demonstrated comparable progression-free survival (PFS) rates with fewer cardiovascular and bleeding side effects compared with ibrutinib in relapsed/refractory CLL/SLL patients (22, 23). Real-world studies further supported these findings, showing lower discontinuation rate in both first-line and relapsed/refractory treatment settings (24), with a better cardiovascular and non-cardiovascular safety profile (25).

BTK is also a validated therapeutic target in mantle cell lymphoma (MCL), especially for older and less physically fit patients (26–31). A Phase II study in patients with relapsed/refractory MCL (after at least two prior lines of therapy) demonstrated that treatment with acalabrutinib achieved high overall (80%) and complete response (40%) rates (32). This study led to accelerated approval of acalabrutinib for MCL by the United States Food and Drug Administration (USFDA) (33, 34). Interim results from the ongoing Phase III ECHO study also showed a statistically significant and clinically meaningful improvement in PFS (66.4 months) with acalabrutinib in combination with standard-of-care chemo-immunotherapy (bendamustine and rituximab) when compared with standard-of-care chemo-immunotherapy alone (49.6 months) in previously untreated adult patients with MCL (35).

Following the USFDA and Central Drugs Standard Control Organization (CDSCO) approval of acalabrutinib for patients with CLL/SLL and MCL (who have received at least one prior therapy) (33, 34), this Phase IV post-marketing study was conducted to assess the safety, clinical outcomes, and quality of life in Indian patients with CLL/SLL and refractory/relapsed MCL who were treated with acalabrutinib.

## 2 Method

### 2.1 Study design

This Phase IV, open-label, single-arm, multi-center, prospective study evaluated the safety and clinical outcomes of acalabrutinib treatment in Indian adult patients with CLL/SLL or MCL. The study was conducted at ten sites in India between July 2021 and May 2023. This study was performed in accordance with the regulatory requirements of the CDSCO, 21 CFR 50, and the New Drugs & Clinical Trials Rules, 2019, and the ethical principles of the Declaration of Helsinki, the International Council for Harmonization (ICH)/Good Clinical Practice (GCP), and other applicable regulatory requirements. Prior to the start of the study, approval was obtained from the independent ethics committee of the participating institutes. The study was prospectively registered with CTRI (CTRI/2021/04/033224) and in the ClinicalTrials.gov registry (NCT04930536). All patients provided written informed consent after which they underwent the following phases during the study: Screening/Enrolment Phase, Treatment Phase, and Follow-up Phase (**Supplementary Figure 1**).

### 2.2 Patients

The key inclusion criteria were: (i) male or female adult patients ≥ 18 years; (ii) patients with CLL/SLL, who were either treatment-naive or have received one or more prior systemic therapy, or MCL, who were relapsed after or were refractory to, one to five previous therapies; (iii) CLL/SLL patients with a confirmed diagnosis of CD20+ CLL that met the International Workshop on Chronic Lymphocytic Leukemia (iwCLL) diagnostic criteria and required therapy by iwCLL 2018 criteria; (iv) MCL patients with confirmed MCL with translocation t(11;14) (q13;q32) or overexpressed cyclin D1 and measurable nodal disease (one or more lesions measuring ≥2 cm in the longest diameter); diagnosis of CLL/SLL was established by local pathology assessment; central pathology review was not performed); (v) adequate hematological function (i.e., (a) absolute neutrophil count (ANC) ≥750 cells/μL or ≥500 cells/μL in patients with documented bone marrow involvement and independent of growth factor support seven days before the assessment; (b) platelet count ≥50,000 cells/μL or ≥30,000 cells/μL in patients with documented bone marrow involvement, and without transfusion support seven days before the assessment); and (vi) adequate liver and kidney function.

The key exclusion criteria were: (i) known prolymphocytic leukemia, central nervous system (CNS) lymphoma or leukemia, or known history of (or currently suspected) Richter’s syndrome; (ii) severe cardiovascular disease such as uncontrolled or symptomatic arrhythmias, congestive heart failure, or myocardial infarction within 6 months of screening, or any Class 3 or 4 cardiac diseases as defined by the New York Heart Association Functional Classification, or QTcB >480 msec at screening; (iii) anticoagulation therapy (e.g., warfarin or equivalent vitamin K antagonists) within 7 days of the first dose of the study drug; (iv) prior exposure to B-cell lymphoma-2 (Bcl-2) inhibitors, other B-cell receptor inhibitors like BTKs; or (v) requiring treatment with proton-pump inhibitors. Refer to the **Supplementary Text Box** for additional eligibility criteria.

### 2.3 Study treatment

Enrolled patients received acalabrutinib capsules 100 mg orally twice daily for six 28-day cycles or until the study drug was discontinued due to either disease progression, unacceptable toxicity, or other reasons, whichever occurred earlier. Patients were followed up for 28 days after the drug discontinuation to collect additional safety data. Dose modifications were allowed to manage adverse events (AEs). Acalabrutinib was temporarily interrupted to manage Common Terminology Criteria for Adverse Events (CTCAE) Grade ≥3 (severe or more) non-hematological treatment-related AEs, Grade 3 thrombocytopenia with significant bleeding, Grade 4 (life-threatening) thrombocytopenia, or Grade 4 neutropenia lasting more than seven days. On resolution of the AEs to ≤Grade 1(mild), acalabrutinib was restarted at the same dosage for the first two occurrences of the AE (i.e., 100 mg twice daily). If the AE recurred for the third time, the dosage was reduced to 100 mg once daily. Thereafter, the treatment was discontinued if the AE recurred a fourth time. The patients were allowed to continue the study drug beyond the study period if they were experiencing clinical benefits as judged by the investigator or had not progressed clinically. Post-study access to the treatment was continued until disease progression, discontinuation due to side effects, or the investigator’s decision.

### 2.4 Assessments

Demographics and baseline characteristics were recorded at baseline, including the International Prognostic Index for Chronic Lymphocytic Leukemia (CLL-IPI), Ann Arbor staging, and Mantle Cell Lymphoma International Prognostic Index (MIPI). The primary objective of the study was to evaluate the safety of acalabrutinib among Indian patients with CLL/SLL or with relapsed/refractory MCL. Safety assessment was conducted from the time of informed consent signature to the end of the study, i.e., throughout the 6-month treatment period and the follow-up period (i.e., 28 days post-treatment period or after the last dose of the study drug in case of early discontinuation of the study drug). Safety endpoints included incidence of adverse events (AEs), treatment-emergent AEs (TEAEs), serious adverse events (SAEs), second primary malignancies, and other AEs of clinical interest, such as atrial fibrillation, anemia, hypertension, infections, and bleeding. AEs were graded by the National Cancer Institute CTCAE version 4.03.

The secondary objective of the study was to assess the clinical outcomes of acalabrutinib based on investigator-assessed objective response rate (ORR). For CLL/SLL, ORR was defined as the proportion of patients achieving either a complete response (CR), partial response (PR), or partial response with lymphocytosis (PRL) during the treatment period, as per the iwCLL 2018 criteria for CLL/SLL (36). For MCL, the Lugano classification for NHL was used (37). Response assessments were performed by the treating investigator at each study site, via computed tomography (CT) or magnetic resonance imaging (MRI), after 3 months and 6 months. No independent central radiology review was conducted in this post-marketing study. Additionally, health-related quality of life was assessed using the EORTC Core Quality of Life Questionnaire (QLQ-C30) at baseline, after 3 months, and after 6 months.

### 2.5 Statistical analysis

No formal sample size calculation was performed, as the primary objective of this study was post-marketing safety surveillance rather than hypothesis testing and the enrolled sample reflects patients treated with acalabrutinib in routine clinical practice during the study period. The study planned to enroll 100 patients and the final number of patients included in the study was those who met the eligibility criteria. The full analysis set (FAS), i.e., patients who received at least one dose of acalabrutinib, was included in the safety and efficacy analysis. Patient demographics, baseline disease characteristics, adverse events, and health-related quality of life were summarized using frequencies and percentages. To quantify uncertainty around the observed adverse event rates, 95% confidence interval (CI) for proportions were computed using the exact method based on the binomial distribution. The sum of CR, PR, and PRL was also summarized using frequencies and percentages, and the 95% CI was calculated using the Clopper-Pearson confidence interval method. All analyses were performed using SAS version 9.4 (SAS Institute Inc., Cary, NC). Given the fixed-duration post-marketing surveillance design, a formal median follow-up and treatment duration were not calculated, as all patients were followed for the same pre-specified duration of six 28-day cycles of treatment or until study drug discontinuation, followed by 28 days of follow-up after the drug discontinuation to collect additional safety data.

## 3 Results

### 3.1 Patient characteristics

**Figure 1:**
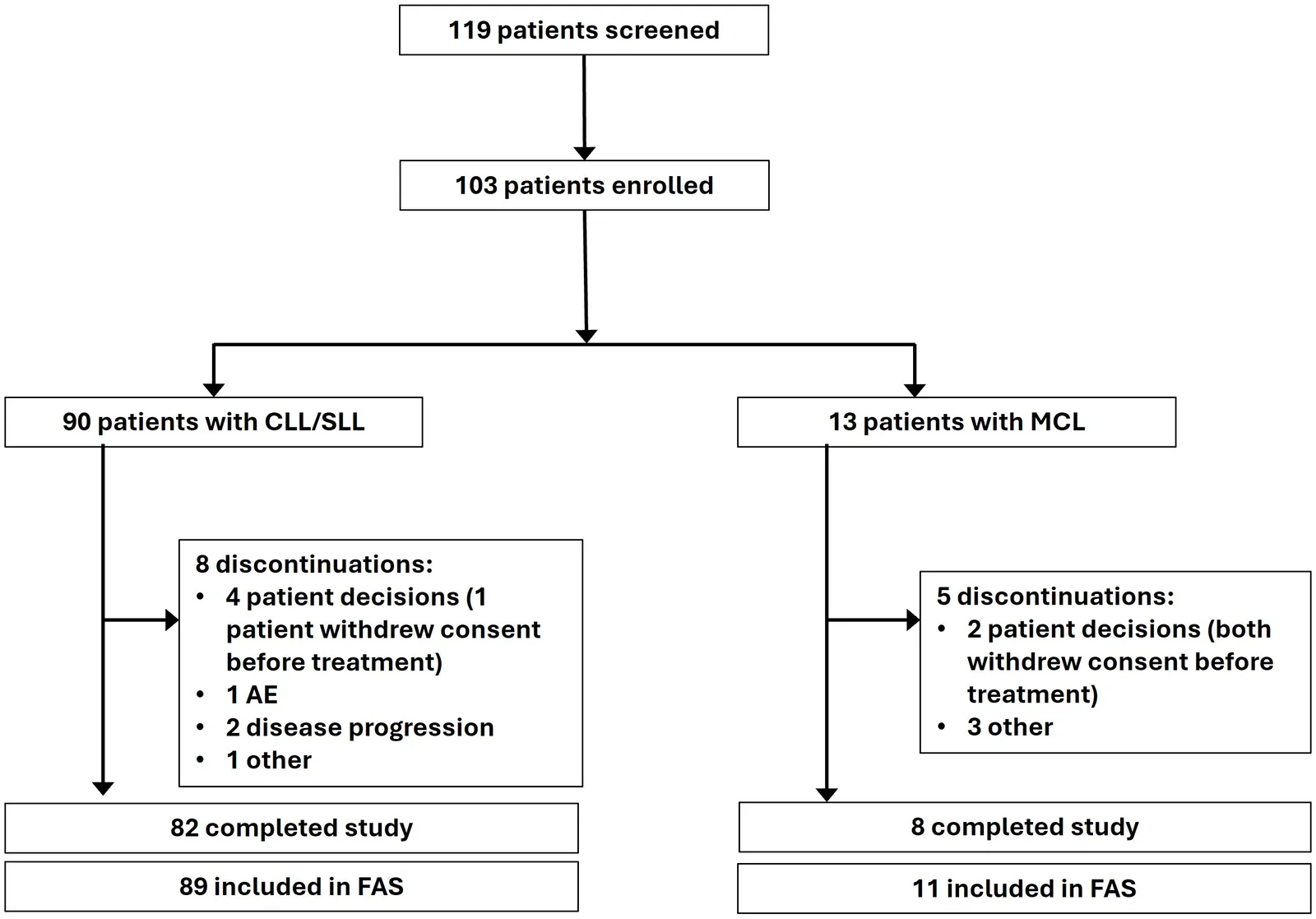
Patient disposition.

The disposition of patients in the study is presented in **Figure 1**. A total of 119 patients were screened, and 103 were enrolled. These included patients with CLL/SLL (n=90) and MCL (n=13). Of these, 100 patients (89 in the CLL/SLL and 11 in the MCL cohort) received at least one dose of acalabrutinib. Ninety (87%) patients completed the study: 82 (91%) in the CLL/SLL cohort and 8 (62%) in the MCL cohort. Thirteen (13%) patients discontinued the study treatment: 8 (9%) in the CLL/SLL cohort and 5 (39%) in the MCL cohort, due to patient decision, adverse events, and other reasons.

Patient demographics and baseline characteristics are presented in **Table 1**. Most patients were males (80%) with a mean age of 61.6 (SD 10.8) years. Among 89 CLL/SLL patients, 74 (83.1%) were treatment-naïve at baseline, indicating that the majority had not received prior disease-directed therapy. However, 15 (16.9%) patients in the CLL/SLL cohort and all 11 (100%) patients in the MCL cohort had received prior therapies, including chemoimmunotherapy such as Rituximab + Cyclophosphamide, Doxorubicin, Vincristine, Prednisone (R-CHOP), Rituximab + Dexamethasone, Cytarabine (Ara-C), Cisplatin (R-DHAP), Rituximab + Cyclophosphamide, Vincristine, Prednisone (R-CVP), R-Benda (Bendamustine + Rituximab), and Rituximab + Cyclophosphamide + Vincristine; chemotherapy such as Cyclophosphamide and Leukeran (Chlorambucil); immunotherapy such as Rituximab and Obinutuzumab; and supportive therapy such as Rasburicase, along with Leukeran + Prednisolone. Other prior/concomitant medications across the overall cohort were dominated by therapies for comorbidity management and supportive care. Frequently reported medications included antidiabetic agents, antihypertensives, lipid-lowering drugs, urate-lowering therapies, anti-infective agents (infection prophylaxis and treatment in an immunocompromised population), analgesics, gastrointestinal agents, respiratory medications, and vitamin supplementation.

**Table 1:** Patient demographics and baseline characteristics in chronic lymphocytic leukemia (cll)/small lymphocytic lymphoma (SLL) and mantle cell lymphoma cohorts (MCL) (FAS).

Demographic and baseline variables	CLL/SLL (N = 89)	MCL (N = 11)	Total (N = 100)
Age (years)			
n	89	11	100
Mean (SD)	61.8 (11.18)	60.3 (6.87)	61.6 (10.77)
Median	64.0	61.0	63.0
(min, max)	(34.00, 86.00)	(52.00, 72.00)	(34.00, 86.00)
Gender, n (%)			
Male	71 (79.78)	9 (81.82)	80 (80.00)
Female	18 (20.22)	2 (18.18)	20 (20.00)
Race, n (%)			
Asian	89 (100)	11 (100)	100 (100)
Chronic lymphocytic leukemia (CLL)/small lymphocytic lymphoma (SLL)			
clinical staging (rai staging), n (%)			
Stage 0	5 (5.62)	–	–
Stage I	12 (13.48)	–	–
Stage II	23 (25.84)	–	–
Stage III	21 (23.60)	–	–
Stage IV	27 (30.34)	–	–
Missing	1 (1.12)	–	–
Disease characteristics, n (%)			
Blood abnormality with lymphadenopathy	54 (60.67)	–	–
Blood abnormality with organomegaly	21 (23.60)	–	–
Only Blood abnormality	13 (14.61)	–	–
Missing	1 (1.12)	–	–
CLL-IPI Score, n(%)			
n	87	–	–
Mean (SD)	4.1 (2.01)	–	–
Median	4.0	–	–
(min, max)	(0.00, 10.00)	–	–
Any genomic alteration observed, n(%)			
Yes	54 (60.67)	–	–
No	35 (39.33)	–	–
If yes			
11q deletion	18 (33.33)	–	–
13q deletion	27 (50.00)	–	–
17p deletion	6 (11.11)	–	–
12 addition	8 (14.81)	–	–
TP53 mutation	13 (24.07)	–	–
IGHV mutation	10 (18.52)	–	–
Others	16 (29.63)	–	–
Mantle cell lymphoma (MCL); n (%)			
Refractory	–	3 (27.27)	–
Relapsed	–	8 (72.73)	–
Clinical Staging (Ann Arbor), n (%)			
Stage I	–	1 (9.09)	–
Stage III	–	3 (27.27)	–
Stage IV	–	7 (63.64)	–
Genetic and molecular features, n (%)			
Overexpressed cyclin D1	–	6 (54.55)	–
Translocation t(11;14) (q13;q32)	–	4 (36.36)	–
Missing	–	1 (9.09)	–
MIPI Score			
n	–	11	–
Mean (SD)	–	6.6 (1.10)	–
Median	–	6.4	–
(min, max)	–	(5.00, 8.60)	–

CLL, chronic lymphocytic leukemia; SLL, small lymphocytic lymphoma; MCL, mantle cell lymphoma; FAS, full analysis set; SD, standard deviation.

- “N” in the column header represents the total number in the FAS.

- “n” in summary statistics represents the total number of patients.

- All Percentage rows are based on the number of patients in each cohort.

- All percentages are computed from the FAS population.

Rai staging at baseline showed that 80% of the patients with CLL/SLL were at Stage II-IV. Most patients presented with blood abnormalities with lymphadenopathy (61%), 24% had organomegaly, and 15% had blood abnormalities without these features. The mean CLL-IPI was 4.1 (SD 2.0), indicating a high-risk patient population (38). Genomic alterations were present in 54 (61%) CLL/SLL patients. Among these, common genetic mutations observed were 13q deletion (50%), 11q deletion (33%), TP53 mutation (24%), and IGHV mutation (19%). Among 11 patients in the MCL cohort, 7 (64%) had Ann Arbor stage IV disease, 6 (54%) had overexpressed cyclin D1, and 4 (36%) had translocation t(11;14) (q13;q32). As this was a post-marketing, real-world study, and all enrolled MCL patients were relapsed/refractory, diagnostic confirmation was based on existing pathology reports (≤6 months old) rather than protocol-mandated repeat testing at the screening visit. Therefore, a lower proportion of MCL patients reporting FISH-confirmed translocation t(11;14) (q13;q32) reflects variability in prior real-world diagnostic workup or missing reports rather than a true absence of the abnormality. The mean MIPI was 6.6 (SD 1.0), indicating a high-risk patient population (39).

### 3.2 Safety

By the end of the follow-up period, 57 (57%) patients reported at least one AE: 48 (54%) in the CLL/SLL cohort and 9 (82%) in the MCL cohort (**Table 2**). According to CTCAE version 4.03, most AEs were mild (Grade 1; 25% in CLL/SLL and 46% in MCL) or moderate (Grade 2; 18% in CLL/SLL and 9% in MCL) in intensity. Severe (Grade 3) AEs were reported in 8 (9%) patients in the CLL/SLL cohort and one (9%) in the MCL cohort. A life-threatening event (Grade 4) was reported in one patient in the CLL/SLL cohort. Overall, three patients experienced Grade 5 events resulting in death: one (1%) patient in the CLL/SLL cohort and two (18%) patients in the MCL cohort. Among AEs of special interest, infections were reported as Grade 3 in 3 (3%) patients (all CLL/SLL) and as Grade 4 in 1 (1%) patient (CLL/SLL), with no Grade 5 infection events reported. Anemia was reported as Grade 3 in 3 (3%) patients (2 CLL/SLL and 1 MCL) and as Grade 4 in 1 (1%) patient (CLL/SLL), with no Grade 5 events. Hemorrhage was reported as a Grade 5 event in 1 (1%) patient in the MCL cohort (fatal subdural hemorrhage), with no Grade 3 or Grade 4 hemorrhagic events. No Grade 3, Grade 4, or Grade 5 hypertension or arrhythmia events were reported.

**Table 2:** Summary of adverse events by severity, causality, and outcome in both cohorts treated with acalabrutinib (FAS).

Adverse events characteristics	CLL/SLL (N = 89)	MCL (N = 11)	Total (N = 100)
	n (%)	n (%)	n (%)
Patients experiencing any adverse event	48 (53.93)	9 (81.82)	57 (57.00)
CTCAE grade			
Mild (Grade 1)	22 (24.72)	5 (45.45)	27 (27.00)
Moderate (Grade 2)	16 (17.98)	1 (9.09)	17 (17.00)
Severe (Grade 3)	8 (8.99)	1 (9.09)	9 (9.00)
Life-threatening or disabling (Grade 4)	1 (1.12)	0	1 (1.00)
Death (Grade 5)	1 (1.12)	2 (18.18)	3 (3.00)
Relationship to investigational product			
Related	13 (14.61)	2 (18.18)	15 (15.00)
Unrelated	35 (39.33)	7 (63.64)	42 (42.00)
Drug withdrawn	3 (3.36)	2 18.18)	5 (5.00)
Outcome			
Recovered without sequelae	27 (30.34)	7 (63.64)	34 (34.00)
Recovered with sequelae	4 (4.49)	0	4 (4.00)
Not recovered/Not resolved	12 (13.48)	0	12 (12.00)
Recovering/resolving	4 (4.49)	0	4 (4.00)
Unknown	1 (1.12)	2 (18.18)	3 (3.00)
Patients experiencing any TEAEs	38 (42.70)	7 (63.64)	45 (45.00)
Patient experiencing any serious TEAEs	8 (8.99)	3 (27.27)	11 (11.00)

CLL, chronic lymphocytic leukemia; CTCAE, Common Terminology Criteria for Adverse Events; FAS, full analysis set; MCL, Mantle cell lymphoma; SLL, small lymphocytic lymphoma;.TEAEs, Treatment-emergent adverse events.

Annotations:

- AEs presented are those reported from the screening visit until 28 days after the last dose of acalabrutinib.

- “N” in the column header represents the total number of patients in the FAS.

- “n” in summary statistics represents the total number of patients in the category.

Most AEs were assessed as unrelated to the study drug (39% in CLL/SLL and 64% in MCL). The majority of the patients recovered from the AEs without sequelae (30% in CLL/SLL and 64% in MCL). Drug-related AEs were reported in 15 (15%) patients: 13 (15%) in the CLL cohort and 2 (18%) in the MCL cohort. Drug discontinuation due to AEs/SAEs was observed in 3 (3.4%) patients in the CLL/SLL cohort and in 2 (18.2%) patients in the MCL cohort. In the CLL/SLL cohort, discontinuations were reported due to two SAEs, including myocardial infarction and COVID-19 pneumonia (event in one patient each), and an AE of elevated lipase levels. In the MCL cohort, both discontinuations were reported due to SAEs, including COVID-19 and dyspnea.

Forty-five (45%; 95% CI: 35.03–55.27) patients reported at least one TEAE: 38 (42.7%; 95% CI: 32.26–53.63) patients in the CLL/SLL cohort and 7 (63.6%; 95% CI: 30.79–89.07) patients in the MCL cohort (**Table 3**). No secondary primary malignancies were reported during the study. Serious TEAEs were reported in 11 (11%; 95% CI: 5.62–18.83) patients: 8 (9%; 95%CI: 3.96–16.95) in CLL/SLL and 3 (27.3%; 95% CI: 6.02–60.97) in the MCL cohort, of which five patients reported serious TEAEs that were possibly related to the drug. These included myocardial infarction, tumor lysis syndrome, and pneumonia in the CLL/SLL cohort, and subdural hemorrhage and dyspnea in the MCL cohort, each reported in one patient. The outcome of adverse events was fatal in three patients, of which death due to subdural hemorrhage and dyspnea (breathlessness) was considered as possibly related to the drug. The most frequently reported TEAEs by preferred term in the CLL/SLL cohort were pyrexia (9%; 95% CI: 3.96–16.95), diarrhea (9%; 95% CI: 3.96–16.95), and cough (5.6%; 95% CI: 1.85–12.63). In the MCL cohort, given the small cohort size (n=11), all TEAEs (by preferred term) occurred in single patients (9.09% each; 95% CI: 0.23–41.28), including thrombocytopenia, abdominal pain, diarrhea, coronavirus infection, rhinitis, subdural hemorrhage, back pain, cough, dyspnea, and hemoptysis.(**Table 3**). Other TEAEs of clinical interest for acalabrutinib reported in the CLL/SLL cohort were infections (13.5%; 95% CI: 7.17–22.37), anemia (5%; 95% CI: 1.24–11.11), hypertension (1%; 95% CI: 0.03–6.10), intracranial hemorrhage (1%; 95% CI: 0.03–6.10), and skin hemorrhage (1%; 95% CI: 0.03–6.10). No reports of arrhythmia (atrial fibrillation) were reported. In the MCL cohort, AEs of clinical interest reported were infections (27.3%; 95%CI: 6.02–60.97) and anemia (9%; 95% CI: 0.23–41.28) (**Table 3**).

**Table 3:** Summary of treatment-emergent AEs by system organ class (SOC) and preferred term (PT) in both cohorts treated with acalabrutinib (FAS).

System organ class (SOC)	CLL/SLL		MCL		Total	
Preferred term (PT)	(N = 89)		(N = 11)		(N = 100)	
	n (%)	95% CI	n (%)	95% CI	n (%)	95% CI
Patient with at least one TEAE	38 (42.70)	(32.26, 53.63)	7 (63.64)	(30.79, 89.07)	45 (45.00)	(35.03, 55.27)
Blood and lymphatic system disorders	7 (7.87)	(3.22, 15.54)	1 (9.09)	(0.23, 41.28)	8 (8.00)	(3.52, 15.16)
Anemia	4 (4.49)	(1.24, 11.11)	1 (9.09)	(0.23, 41.28)	5 (5.00)	(1.64, 11.28)
Leukocytosis	2 (2.25)	(0.27, 7.88)			2 (2.00)	(0.24, 7.04)
Lymphadenopathy	1 (1.12)	(0.03, 6.10)			1 (1.00)	(0.03, 5.45)
Lymphocytosis	1 (1.12)	(0.03, 6.10)			1 (1.00)	(0.03, 5.45)
Thrombocytopenia			1 (9.09)	(0.23, 41.28)	1 (1.00)	(0.03, 5.45)
Cardiac disorders	3 (3.37)	(0.70, 9.54)			3 (3.00)	(0.62, 8.52)
Angina pectoris	1 (1.12)	(0.03, 6.10)			1 (1.00)	(0.03, 5.45)
Conduction disorder	1 (1.12)	(0.03, 6.10)			1 (1.00)	(0.03, 5.45)
Myocardial infarction	2 (2.25)	(0.27, 7.88)			2 (2.00)	(0.24, 7.04)
Ear and labyrinth disorders	1 (1.12)	(0.03, 6.10)			1 (1.00)	(0.03, 5.45)
Deafness	1 (1.12)	(0.03, 6.10)			1 (1.00)	(0.03, 5.45)
Eye disorders	1 (1.12)	(0.03, 6.10)			1 (1.00)	(0.03, 5.45)
Cataract	1 (1.12)	(0.03, 6.10)			1 (1.00)	(0.03, 5.45)
Gastrointestinal disorders	14 (15.73)	(8.88, 24.98)	2 (18.18)	(2.28, 51.78)	16 (16.00)	(9.43, 24.68)
Abdominal pain upper	1 (1.12)	(0.03, 6.10)	1 (9.09)	(0.23, 41.28)	2 (2.00)	(0.24, 7.04)
Constipation	4 (4.49)	(1.24, 11.11)			4 (4.00)	(1.10, 9.93)
Diarrhea	8 (8.99)	(3.96, 16.95)	1 (9.09)	(0.23, 41.28)	9 (9.00)	(4.20, 16.40)
Gastritis	1 (1.12)	(0.03, 6.10)			1 (1.00)	(0.03, 5.45)
Gastroesophageal reflux disease	1 (1.12)	(0.03, 6.10)			1 (1.00)	(0.03, 5.45)
Hemorrhoidal hemorrhage	1 (1.12)	(0.03, 6.10)			1 (1.00)	(0.03, 5.45)
Hemorrhoids	1 (1.12)	(0.03, 6.10)			1 (1.00)	(0.03, 5.45)
Nausea	2 (2.25)	(0.27, 7.88)			2 (2.00)	(0.24, 7.04)
Stomatitis	1 (1.12)	(0.03, 6.10)			1 (1.00)	(0.03, 5.45)
General disorders and administration site conditions	16 (17.98)	(10.64, 27.55)			16 (16.00)	(9.43, 24.68)
Asthenia	1 (1.12)	(0.03, 6.10)			1 (1.00)	(0.03, 5.45)
Face oedema	1 (1.12)	(0.03, 6.10)			1 (1.00)	(0.03, 5.45)
Fatigue	2 (2.25)	(0.27, 7.88)			2 (2.00)	(0.24, 7.04)
Inflammation	1 (1.12)	(0.03, 6.10)			1 (1.00)	(0.03, 5.45)
Malaise	1 (1.12)	(0.03, 6.10)			1 (1.00)	(0.03, 5.45)
Oedema peripheral	3 (3.37)	(0.70, 9.54)			3 (3.00)	(0.62, 8.52)
Peripheral swelling	1 (1.12)	(0.03, 6.10)			1 (1.00)	(0.03, 5.45)
Pyrexia	8 (8.99)	(3.96, 16.95)			8 (8.00)	(3.52, 15.16)
Immune system disorders	2 (2.25)	(0.27, 7.88)			2 (2.00)	(0.24, 7.04)
Hypersensitivity	2 (2.25)	(0.27, 7.88)			2 (2.00)	(0.24, 7.04)
Infections and infestations	12 (13.48)	(7.17, 22.37)	3 (27.27)	(6.02, 60.97)	15 (15.00)	(8.65, 23.53)
Abdominal infection	1 (1.12)	(0.03, 6.10)			1 (1.00)	(0.03, 5.45)
Bacterial infection	2 (2.25)	(0.27, 7.88)			2 (2.00)	(0.24, 7.04)
COVID-19 pneumonia	1 (1.12)	(0.03, 6.10)			1 (1.00)	(0.03, 5.45)
Coronavirus infection			1 (9.09)	(0.23, 41.28)	1 (1.00)	(0.03, 5.45)
Dengue fever	1 (1.12)	(0.03, 6.10)			1 (1.00)	(0.03, 5.45)
Fungal infection	1 (1.12)	(0.03, 6.10)			1 (1.00)	(0.03, 5.45)
Furuncle	1 (1.12)	(0.03, 6.10)			1 (1.00)	(0.03, 5.45)
Gastrointestinal infection	1 (1.12)	(0.03, 6.10)			1 (1.00)	(0.03, 5.45)
Oral herpes	1 (1.12)	(0.03, 6.10)			1 (1.00)	(0.03, 5.45)
Pneumonia	2 (2.25)	(0.27, 7.88)			2 (2.00)	(0.24, 7.04)
Respiratory tract infection viral	1 (1.12)	(0.03, 6.10)			1 (1.00)	(0.03, 5.45)
Rhinitis			1 (9.09)	(0.23, 41.28)	1 (1.00)	(0.03, 5.45)
Skin infection	1 (1.12)	(0.03, 6.10)			1 (1.00)	(0.03, 5.45)
Upper respiratory tract infection	1 (1.12)	(0.03, 6.10)	1 (9.09)	(0.23, 41.28)	2 (2.00)	(0.24, 7.04)
Urinary tract infection	2 (2.25)	(0.27, 7.88)			2 (2.00)	(0.24, 7.04)
Viral upper respiratory tract infection	2 (2.25)	(0.27, 7.88)			2 (2.00)	(0.24, 7.04)
Injury, poisoning, and procedural complications	1 (1.12)	(0.03, 6.10)	1 (9.09)	(0.23, 41.28)	2 (2.00)	(0.24, 7.04)
Contusion	1 (1.12)	(0.03, 6.10)			1 (1.00)	(0.03, 5.45)
Subdural hemorrhage			1 (9.09)	(0.23, 41.28)	1 (1.00)	(0.03, 5.45)
Investigations	3 (3.37)	(0.70, 9.54)	1 (9.09)	(0.23, 41.28)	4 (4.00)	(1.10, 9.93)
Blood glucose increased	1 (1.12)	(0.03, 6.10)			1 (1.00)	(0.03, 5.45)
Lipase	1 (1.12)	(0.03, 6.10)			1 (1.00)	(0.03, 5.45)
Platelet count decreased			1 (9.09)	(0.23, 41.28)	1 (1.00)	(0.03, 5.45)
SARS-CoV-2 test positive	1 (1.12)	(0.03, 6.10)			1 (1.00)	(0.03, 5.45)
Metabolism and nutrition disorders	4 (4.49)	(1.24, 11.11)			4 (4.00)	(1.10, 9.93)
Decreased appetite	1 (1.12)	(0.03, 6.10)			1 (1.00)	(0.03, 5.45)
Hypovitaminosis	1 (1.12)	(0.03, 6.10)			1 (1.00)	(0.03, 5.45)
Hypovolemia	1 (1.12)	(0.03, 6.10)			1 (1.00)	(0.03, 5.45)
Iron deficiency	1 (1.12)	(0.03, 6.10)			1 (1.00)	(0.03, 5.45)
Metabolic acidosis	1 (1.12)	(0.03, 6.10)			1 (1.00)	(0.03, 5.45)
Musculoskeletal and connective tissue disorders	5 (5.62)	(1.85, 12.63)	1 (9.09)	(0.23, 41.28)	6 (6.00)	(2.23, 12.60)
Arthralgia	2 (2.25)	(0.27, 7.88)			2 (2.00)	(0.24, 7.04)
Back pain	2 (2.25)	(0.27, 7.88)	1 (9.09)	(0.23, 41.28)	3 (3.00)	(0.62, 8.52)
Pain in extremity	1 (1.12)	(0.03, 6.10)			1 (1.00)	(0.03, 5.45)
Nervous system disorders	4 (4.49)	(1.24, 11.11)			4 (4.00)	(1.10, 9.93)
Hemorrhage intracranial	1 (1.12)	(0.03, 6.10)			1 (1.00)	(0.03, 5.45)
Headache	3 (3.37)	(0.70, 9.54)			3 (3.00)	(0.62, 8.52)
Psychiatric disorders	1 (1.12)	(0.03, 6.10)			1 (1.00)	(0.03, 5.45)
Insomnia	1 (1.12)	(0.03, 6.10)			1 (1.00)	(0.03, 5.45)
Renal and urinary disorders	2 (2.25)	(0.27, 7.88)			2 (2.00)	(0.24, 7.04)
Dysuria	1 (1.12)	(0.03, 6.10)			1 (1.00)	(0.03, 5.45)
Hematuria	1 (1.12)	(0.03, 6.10)			1 (1.00)	(0.03, 5.45)
Respiratory, thoracic, and mediastinal disorders	8 (8.99)	(3.96, 16.95)	3 (27.27)	(6.02, 60.97)	11 (11.00)	(5.62, 18.83)
Chronic obstructive pulmonary disease	1 (1.12)	(0.03, 6.10)			1 (1.00)	(0.03, 5.45)
Cough	5 (5.62)	(1.85, 12.63)	1 (9.09)	(0.23, 41.28)	6 (6.00)	(2.23, 12.60)
Dyspnea	1 (1.12)	(0.03, 6.10)	1 (9.09)	(0.23, 41.28)	2 (2.00)	(0.24, 7.04)
Hemoptysis	1 (1.12)	(0.03, 6.10)	1 (9.09)	(0.23, 41.28)	2 (2.00)	(0.24, 7.04)
Oropharyngeal pain	1 (1.12)	(0.03, 6.10)			1 (1.00)	(0.03, 5.45)
Skin and subcutaneous tissue disorders	7 (7.87)	(3.22, 15.54)			7 (7.00)	(2.86, 13.89)
Ecchymosis	1 (1.12)	(0.03, 6.10)			1 (1.00)	(0.03, 5.45)
Hair color changes	1 (1.12)	(0.03, 6.10)			1 (1.00)	(0.03, 5.45)
Petechiae	1 (1.12)	(0.03, 6.10)			1 (1.00)	(0.03, 5.45)
Pruritus	2 (2.25)	(0.27, 7.88)			2 (2.00)	(0.24, 7.04)
Rash maculo-papular	2 (2.25)	(0.27, 7.88)			2 (2.00)	(0.24, 7.04)
Skin hemorrhage	1 (1.12)	(0.03, 6.10)			1 (1.00)	(0.03, 5.45)
Skin lesion	1 (1.12)	(0.03, 6.10)			1 (1.00)	(0.03, 5.45)
Vascular disorders	4 (4.49)	(1.24, 11.11)			4 (4.00)	(1.10, 9.93)
Hypertension	1 (1.12)	(0.03, 6.10)			1 (1.00)	(0.03, 5.45)
Hypotension	1 (1.12)	(0.03, 6.10)			1 (1.00)	(0.03, 5.45)
Pallor	1 (1.12)	(0.03, 6.10)			1 (1.00)	(0.03, 5.45)
Thrombosis	1 (1.12)	(0.03, 6.10)			1 (1.00)	(0.03, 5.45)

CI, Confidence Interval; CLL, chronic lymphocytic leukemia; FAS, full analysis set; MCL, Mantle cell lymphoma; SLL, small lymphocytic lymphoma; TEAE, Treatment-emergent adverse.

- The safety assessment period was from Screening to 28 days after the last cycle.

- “N” in the column header represents the total number of patients in the FAS.

- “n” in summary statistics represents the total number of patients with TEAE.

- 95% CI was obtained using exact method on binominal distribution.

### 3.3 Clinical outcomes

#### 3.3.1 Objective response rate

**Figure 2:**
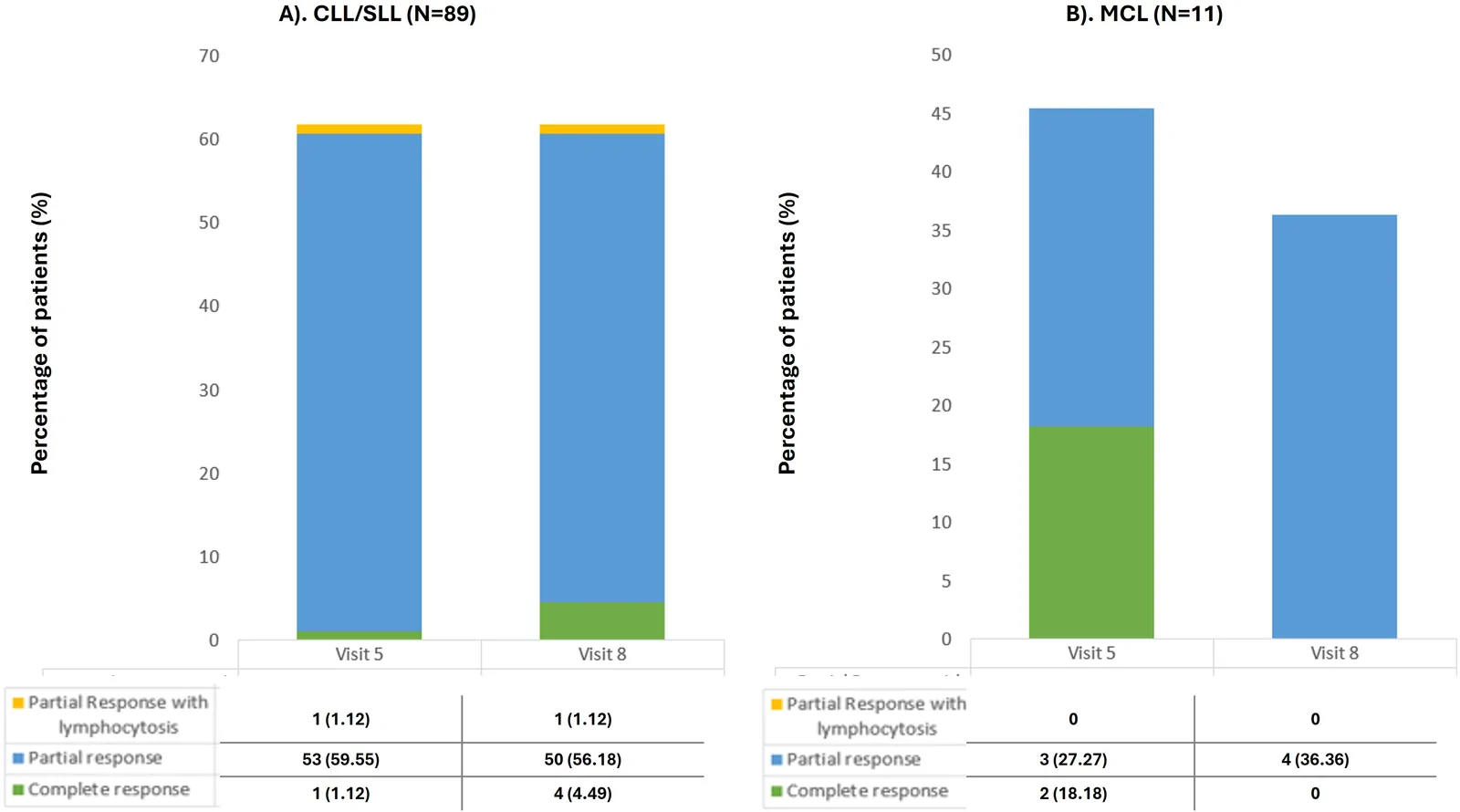
Comparative objective response rate after 3 cycles of acalabrutinib (visit 5) and 6 cycles of acalabrutinib (EOT - visit 8) (FAS). **(A)** CLL/SLL cohort; **(B)** MCL cohort. CLL, chronic lymphocytic leukemia; EOT, End of treatment; FAS, full analysis set; MCL, Mantle cell lymphoma; N, total number of patients in the cohort; SLL, small lymphocytic lymphoma.

In patients with CLL/SLL (n=89), the ORR was 62% (95% CI: 50.9–71.9); 5% achieved a complete response, 56% achieved a partial response, and 1% achieved a partial response with lymphocytosis (**Figure 2**) during the follow-up period. In patients with MCL (n=11), the ORR was 36% (95% CI: 10.9–69.2); all patients achieved a partial response to the treatment.

#### 3.3.2 Health-related quality of life

At baseline, the mean total score on the EORTC Core Quality of Life Questionnaire (QLQ-C30) was 56.9 (SD 12.5) in the CLL/SLL cohort and 57.0 (SD 6.5) in the MCL cohort. After 6 months, the mean total score was 57.9 (SD 9.3) and 58.6 (SD 6.8) in the CLL/SSL and MCL cohorts, respectively (**Supplementary Table 1**).

## 4 Discussion

This study, conducted in India, evaluated the safety and clinical outcomes of acalabrutinib in patients diagnosed with either CLL/SLL or refractory/relapsed MCL. Acalabrutinib demonstrated a safety profile consistent with the established safety profile of the drug (16, 22, 23, 40). The ORR observed with acalabrutinib was consistent with another real-world study (41).

The AEs reported in this study were consistent with those previously identified with acalabrutinib in Phase III studies (16, 22, 23, 40) and in real-world studies (42). No new safety signals were identified. The overall TEAE rate of 45% (95% CI: 35.03–55.27) in our study was numerically lower than previously reported rates, likely due to differences in follow-up duration, patient selection, and the real-world setting, where patients with significant comorbidities may have been managed differently. The serious TEAE rate of 11% (95% CI: 5.62–18.83) and the occurrence of drug-related TEAEs/SAEs are in line with the known risks of BTK inhibitors (43, 44). Notably, the two fatal events considered possibly drug-related (subdural hemorrhage and dyspnea) and other isolated events of hemorrhages (intracranial, hemorrhoidal, and skin) and respiratory disorders highlight the importance of vigilant monitoring for hemorrhagic and pulmonary complications in clinical practice. The MCL cohort demonstrated a higher proportion of AEs compared with the CLL/SLL cohort, including any-grade AEs (81.8% vs 53.9%) and deaths (18.2% vs 1.1%). However, these findings should be interpreted with caution, given the small sample size of the MCL cohort (n=11), which can disproportionately influence percentage estimates. Additionally, the MCL population represented a clinically high-risk group, characterized predominantly by advanced-stage disease (64% with Ann Arbor Stage IV) and elevated mean MIPI scores (6.6 ± 1.10). These are the factors associated with poorer outcomes. No secondary primary malignancies were observed in this study, possibly due to the short follow-up, which may limit the ability to detect such events.

Cardiovascular events are the most commonly reported adverse events with first-generation BTK inhibitors such as ibrutinib. Arrhythmias (atrial fibrillation) and hypertension were pre-specified AEs of clinical interest (AESIs) in this study. Atrial fibrillation was not reported, and hypertension was reported in 1 (1.12%) patient in the CLL/SLL cohort, consistent with the low incidence reported in another real-world study on acalabrutinib (1.6%) (42). Acalabrutinib’s greater selectivity for BTK and reduced off-target kinase inhibition have been proposed as the mechanisms underlying its more favorable cardiovascular tolerability, and our findings are consistent with this (22, 40). The low incidences of other non-pre-specified cardiovascular events were also reported, including one serious event of myocardial infarction. However, these findings should be interpreted with caution, as severe cardiovascular disease was an exclusion criterion of the study. Additionally, given the limited sample size (n=100) and short follow-up period (6 months), the ability to detect infrequent or late-onset cardiovascular events remains limited.

The observed rates of infections in this study (13% in the CLL/SLL cohort and 27% in the MCL cohort) were consistent with those observed in other studies of CLL/SLL and MCL patients receiving acalabrutinib therapy (16, 22) and are attributed to the underlying immunosuppressive nature of CLL/SLL and MCL. Overall, while the type of TEAEs observed aligned with the known safety profile of acalabrutinib, the frequency of these AEs reported in this study was lower than in other real-world and phase III studies, likely due to the short duration of this study (34, 45).

In this 6-month study, all patients were intended to receive acalabrutinib for six 28-day cycles (168 days), per the fixed-duration design of this post-marketing surveillance study; and a formal median duration of treatment was not calculated. The rate of discontinuation of drug due to AEs was low (3.6% in the CLL/SLL cohort and 18.2% in the MCL cohort), indicating that acalabrutinib was well-tolerated, and the majority of the patients continued the study treatment for the full planned treatment duration. The rate of discontinuation due to AEs observed in previously reported phase III studies varied from 9% to 15% in treatment-naïve or relapsed/refractory CLL/SLL (16, 22, 23, 40, 46) over a median follow-up ranging from 16 to 47 months, and 6% in relapsed/refractory MCL, over 15 months of follow-up (32). A pooled analysis of studies on acalabrutinib in CLL/SLL patients showed an overall discontinuation rate of 17% in patients over a median follow-up of 44 months (47). A real-world study showed a discontinuation rate of 20% in CLL/SLL patients over 12 months of follow-up, with more than half of the patients discontinuing due to adverse events (24). The lower discontinuation rate reported in this study may be due to the study’s short duration, which might not have captured the full spectrum of AEs that could lead to discontinuation. Additionally, discontinuation due to myocardial infarction and COVID-19 pneumonia that were assessed as drug-related in the CLL/SLL cohort were infrequent and did not represent a class-defining toxicity pattern. Although in the MCL cohort, dyspnea assessed as drug-related warrants monitoring in future studies. Overall, the low discontinuation rates and AE-related discontinuation patterns observed across both cohorts were manageable and consistent with the known safety profile of the drug class, supporting the continued clinical evaluation of this agent in both indications.

This study also evaluated the clinical response of acalabrutinib in CLL/SLL and refractory/relapsed MCL patients. The ORR in the CLL/SLL and MCL cohorts was 62% and 36%, respectively. Most patients included in the study were at an advanced stage of the disease, indicated by the Rai and Ann Arbor classification, and presented with high-risk genomic mutations (i.e., TP53 mutations, 17p deletions, unmutated IGHV genes, 13q deletions, and 11q deletions). The overall response seen in this study population was consistent with another real-world study (41). Response rates were lower than those reported in other Phase II/III studies that included patients with similar high-risk disease characteristics (16, 22, 32, 40, 48). This variation in treatment response could be due to the less controlled settings and diverse populations typically seen in real-world studies, compared with the controlled conditions of phase II/III studies. Furthermore, the study had a shorter follow-up period than Phase III trials, which not only precluded a definitive assessment of response durability but may also have led to an underestimation of the best overall response. BTK inhibitor–based responses are known to deepen over time, with many patients achieving deeper responses beyond 6 months of therapy (32). Similarly, the ORR of 36% observed in the MCL cohort is notably lower than the ~80% reported in pivotal acalabrutinib trials; however, given the very small sample size of 11 patients, which is reflected in the wide 95% confidence interval (10.93–69.21), and the severity of disease in enrolled MCL patients, makes the ORR estimate highly sensitive to individual patient outcomes. Therefore, the ORR reported here should be interpreted as a preliminary estimate, and longer follow-up will be necessary to fully characterize response depth and durability in this cohort.

In the context of BTK inhibitor therapy, patients may remain on treatment despite not meeting formal response criteria, particularly during the early phases of therapy. This is partly explained by the well-recognized phenomenon of treatment-emergent lymphocytosis, especially in CLL/SLL, in which lymphocytes transiently redistribute from lymphoid tissues into the peripheral blood. As a result, conventional iwCLL 2018 response criteria may underestimate early clinical benefit (36, 49). Consistent with this mechanism, patients with stable disease or early clinical benefit are often maintained on therapy in clinical practice, with the expectation that the response will deepen over time. However, in the present study, response assessments between Visit 5 (after 3 months of treatment) and Visit 8 (at the end of 6 months of treatment) showed largely stable ORR without clear evidence of further deepening. At the same time, the overall discontinuation rate remained low, with discontinuations primarily driven by patient decision, adverse events, and disease progression. This suggests that a proportion of patients continued therapy with disease control that did not translate into formal response improvement during the observation period. Taken together, these findings indicate that while stable disease may represent ongoing clinical benefit in this real-world population, particularly in high-risk CLL/SLL and MCL, the absence of clear response deepening highlights the need for cautious interpretation. A more granular evaluation of response kinetics and treatment duration would further clarify the relationship between sustained therapy and clinical outcomes. Nonetheless, the results of the present study provide valuable data on the clinical response of acalabrutinib in Indian patients with CLL/SLL and MCL in a real-world setting. Besides clinical response, changes in health-related quality of life were also evaluated during the treatment with Acalabrutinib. The scores were maintained, indicating that the patient’s quality of life did not deteriorate during treatment.

Several limitations of this study warrant acknowledgment. First, the absence of a formal sample size calculation limits the statistical power for efficacy comparisons; however, this is consistent with the post-marketing surveillance design of the study, which was not intended for hypothesis testing. Second, the treatment duration of approximately six months is shorter than the continuous administration schedule typical of BTK inhibitor therapy in clinical practice. This limits the assessment of response durability (given time-dependent response kinetics), precludes the detection of late-onset adverse events, and may have led to an underestimation of the best overall response, as BTK inhibitor responses are known to deepen with prolonged, continuous exposure. Another limitation was that, due to the modest sample size, subgroup analyses stratified by individual genomic features such as TP53 mutations, 17p deletions, unmutated IGHV genes, 13q deletions, and 11q deletions were not feasible, as these would have yielded cell sizes too small for meaningful interpretation. Future larger real-world studies are warranted to evaluate the prognostic impact of these features on acalabrutinib outcomes in Indian patients. Additionally, individual patient-level follow-up and dosing data were not available to calculate formal median follow-up and treatment duration; however, all patients were followed for the pre-specified study duration inherent to the post-marketing surveillance framework. As a result, the exact relationship between individual treatment exposure and the timing of responses or adverse events could not be precisely characterized, and the reported outcomes should be interpreted in the context of the fixed six-month protocol duration rather than actual patient-level treatment duration. Nevertheless, the real-world design provides complementary and clinically relevant evidence on the safety and tolerability of acalabrutinib in routine practice, where patient heterogeneity, comorbidity burden, and treatment decisions more closely reflect the broader population. Future studies with larger cohorts and prospective pharmacovigilance frameworks are warranted to better characterize the relationship between specific adverse events and dose modification patterns in both CLL/SLL and MCL.

## 5 Conclusion

This real-world study provides valuable evidence supporting the safety, tolerability, and effectiveness of acalabrutinib in Indian patients with CLL/SLL and MCL. The adverse event profile was manageable, with low rates of treatment discontinuation, and no new safety signals were observed beyond those previously reported in global clinical trials. Promising overall response (efficacy outcomes) observed across both cohorts further supports the clinical utility of acalabrutinib in routine practice within Indian patients.

## Data Availability

The datasets presented in this study can be found in online repositories. The names of the repository/repositories and accession number(s) can be found below: https://clinicaltrials.gov/study/NCT04930536?cond=NCT04930536&rank=1.

## References

[1] Lad D Malhotra P Varma N Sachdeva MS Das A Srinivasan R . CLL: Common leukemia; uncommon presentations. Indian J Hematol Blood Transfusion. (2016) 32:268–75. doi: 10.1007/s12288-014-0488-8 27429518 PMC4930746

[2] Lad DP Malhotra P Varma S . Chronic lymphocytic leukemia: Inception to cure: Are we there? Indian J Hematol Blood Transfusion. (2013) 29:1–10. doi: 10.1007/s12288-012-0192-5 24426325 PMC3572254

[3] Lad DP Tejaswi V Jindal N Malhotra P Khadwal A Prakash G . Modified CLL International Prognostic Index (CLL-LIPI) using lymphocyte doubling time (LDT) in place of IgHV mutation status in resource-limited settings predicts time to first treatment and overall survival. Leukemia Lymphoma. (2020) 61:1512–5. doi: 10.1080/10428194.2020.1719099 31992098

[4] Tejaswi V Lad DP Jindal N Prakash G Malhotra P Khadwal A . Chronic lymphocytic leukemia: Real-world data from India. JCO Global Oncol. (2020) 6:866–72. doi: 10.1200/go.20.00032 32579486 PMC7328099

[5] Jindal N Lad DP Malhotra P Prakash G Khadwal A Jain A . Randomized controlled trial of individualized, low dose, fixed duration lenalidomide maintenance versus observation after frontline chemo-immunotherapy in CLL. Leukemia Lymphoma. (2021) 62:1674–81. doi: 10.1080/10428194.2021.1885668 33612059

[6] Malhotra P Hogan WJ Litzow MR Elliott MA Gastineau DA Ansell SM . Long-term outcome of allogeneic stem cell transplantation in chronic lymphocytic leukemia: Analysis after a minimum follow-up of 5 years. Leukemia Lymphoma. (2008) 49:1724–30. doi: 10.1080/10428190802263535 18798106

[7] Eichhorst B Ghia P Bosch F Clifford R Gregor M Guieze R . EHA guidelines on management of chronic lymphocytic leukemia and Richter transformation. HemaSphere. (2026) 10:e70403. doi: 10.1002/hem3.70403 42293463 PMC13254007

[8] Lad DP Malhotra P Khadwal A Prakash G Jain A Varma S . Reduced dose ibrutinib due to financial toxicity in CLL. Indian J Hematol Blood Transfusion. (2019) 35:260–4. doi: 10.1007/s12288-018-1011-4 30988561 PMC6439022

[9] Nehra P Chauhan AS Malhotra P Kumar L Singh A Gupta N . Cost-effectiveness analysis of different combination therapies for the treatment of chronic lymphocytic leukaemia in India. Lancet Regional Health - Southeast Asia. (2023) 13:100201. doi: 10.1016/j.lansea.2023.100201 37383548 PMC10305972

[10] Banerji V Aw A Robinson S Doucette S Christofides A Sehn LH . Bruton tyrosine kinase inhibitors for the frontline treatment of chronic lymphocytic leukemia. Curr Oncol. (2020) 27:645–55. doi: 10.3747/co.27.6795 33380880 PMC7755444

[11] Schmelz J Heesen P Patnaik A Holder T Lee HJ Molony DA . Bruton tyrosine kinase inhibitors for chronic lymphocytic leukemia. Cochrane Database Systematic Rev. (2021) 2021(9):CD014681. doi: 10.1002/14651858.cd01468142587375

[12] Eichhorst B Robak T Montserrat E Ghia P Niemann CU Kater AP . Chronic lymphocytic leukaemia: ESMO clinical practice guidelines for diagnosis, treatment and follow-up. Ann Oncol. (2021) 32:23–33. doi: 10.1093/annonc/mdq180 33091559

[13] Wierda WG Brown J Abramson JS Awan F Bilgrami SF Bociek G . NCCN Guidelines® Insights: Chronic lymphocytic leukemia/small lymphocytic lymphoma, Version 3.2022: Featured updates to the NCCN Guidelines. J Natl Compr Cancer Network. (2022) 20:622–34. doi: 10.6004/jnccn.2019.0002 35714675

[14] Burger JA Barr PM Robak T Owen C Ghia P Tedeschi A . Long-term efficacy and safety of first-line ibrutinib treatment for patients with CLL/SLL: 5 years of follow-up from the phase 3 RESONATE-2 study. Leukemia. (2020) 34:787–98. doi: 10.1038/s41375-019-0602-x 31628428 PMC7214263

[15] Byrd JC Hillmen P O’Brien S Barrientos JC Reddy NM Coutre S . Long-term follow-up of the RESONATE phase 3 trial of ibrutinib vs ofatumumab. Blood. (2019) 133:2031–42. doi: 10.1182/blood-2018-08-870238 30842083 PMC6509542

[16] Ghia P Pluta A Wach M Lysak D Kozak T Simkovic M . ASCEND: Phase III, randomized trial of acalabrutinib versus idelalisib plus rituximab or bendamustine plus rituximab in relapsed or refractory chronic lymphocytic leukemia. J Clin Oncol. (2020) 38:2849–61. doi: 10.1200/jco.19.03355 32459600

[17] Shaw ML . Second-generation BTK inhibitors hit the treatment bullseye with fewer off-target effects. Am J Managed Care. (2020) 26:SP226–SP7. doi: 10.37765/ajmc.2020.88475 32840975

[18] Tam C Thompson PA . BTK inhibitors in CLL: Second-generation drugs and beyond. Blood Adv. (2024) 8:2300–9. doi: 10.1182/bloodadvances.2023012221 38478390 PMC11117011

[19] Stephens DM . Second-generation Bruton's tyrosine kinase inhibitors: Simply the best treatments for chronic lymphocytic leukemia? J Clin Oncol. (2021) 39:3419–22. doi: 10.1200/jco.21.01414 34310198 PMC8547933

[20] Barf T Covey T Izumi R Van De Kar B Gulrajani M Van Lith B . Acalabrutinib (ACP-196): A covalent Bruton tyrosine kinase inhibitor with a differentiated selectivity and *in vivo* potency profile. J Pharmacol Exp Ther. (2017) 363:240–52. doi: 10.1124/jpet.117.242909 28882879

[21] Wu J Zhang M Liu D . Acalabrutinib (ACP-196): A selective second-generation BTK inhibitor. J Hematol Oncol. (2016) 9:21. doi: 10.1186/s13045-016-0250-9 26957112 PMC4784459

[22] Byrd JC Hillmen P Ghia P Kater AP Chanan-Khan A Furman RR . Acalabrutinib versus ibrutinib in previously treated chronic lymphocytic leukemia: Results of the first randomized phase III trial. J Clin Oncol. (2021) 39:3441–52. doi: 10.1200/jco.21.01210 34310172 PMC8547923

[23] Seymour JF Byrd JC Ghia P Kater AP Chanan-Khan A Furman RR . Detailed safety profile of acalabrutinib vs ibrutinib in previously treated chronic lymphocytic leukemia in the ELEVATE-RR trial. Blood. (2023) 142:687–99. doi: 10.1182/blood.2022018818 37390310 PMC10644206

[24] Roeker LE DerSarkissian M Ryan K Chen Y Duh MS Wahlstrom SK . Real-world comparative effectiveness of acalabrutinib and ibrutinib in patients with chronic lymphocytic leukemia. Blood Adv. (2023) 7:4291–301. doi: 10.1182/bloodadvances.2023009739 37163361 PMC10424141

[25] Nunes RAB Avezum Á De Oliveira Marques M Baiocchi OCCG Bachour P . Three-year cardiovascular and non-cardiovascular adverse events in patients with chronic lymphocytic leukemia or small cell lymphocytic lymphoma treated with Bruton tyrosine kinase inhibitors acalabrutinib or ibrutinib: A real-world analysis. Ann Hematol. (2024) 103:4613–20. doi: 10.2139/ssrn.4697842 39153144

[26] Eyre TA Bishton MJ McCulloch R O'Reilly M Sanderson R Menon G . Diagnosis and management of mantle cell lymphoma: A British Society for Haematology guideline. Br J Haematol. (2024) 204:108–26. doi: 10.1111/bjh.19131 37880821

[27] McKay P Leach M Jackson B Robinson S Rule S . Guideline for the management of mantle cell lymphoma. Br J Haematol. (2018) 182:46–62. doi: 10.1111/bjh.15283 29767454

[28] Yoon DH Cao J Chen T-Y Izutsu K Kim SJ Kwong YL . Treatment of mantle cell lymphoma in Asia: A consensus paper from the Asian Lymphoma Study Group. J Hematol Oncol. (2020) 13:21. doi: 10.1186/s13045-020-00855-9 32183871 PMC7079508

[29] Girard J Reneau J Devata S Wilcox RA Kaminski MS Mercer J . Evaluating acalabrutinib in the treatment of mantle cell lymphoma: Design, development, and place in therapy. OncoTargets Ther. (2019) 12:8003–14. doi: 10.2147/ott.s155778 31686856 PMC6777435

[30] Martin P Maddocks K Leonard JP Ruan J Goy A Wagner-Johnston N . Postibrutinib outcomes in patients with mantle cell lymphoma. Blood. (2016) 127:1559–63. doi: 10.1182/blood-2015-10-673145 26764355

[31] Avyakta Kallam Md JMV . Current treatments in mantle cell lymphoma. ONCOLOGY. (2023) 3708:326–33. doi: 10.46883/2023.25921002 37616519

[32] Wang M Rule S Zinzani PL Goy A Casasnovas O Smith SD . Acalabrutinib in relapsed or refractory mantle cell lymphoma (ACE-LY-004): A single-arm, multicentre, phase 2 trial. Lancet. (2018) 391:659–67. doi: 10.1016/s0140-6736(17)33108-2 29241979 PMC7864374

[33] Calquence® . Abbreviated prescribing information, in acalabrutinib 100mg capsules. AstraZeneca, (2022).

[34] Acalabrutinib . CALQUENCE (Acalabrutinib) Tablets, for Oral Use. Acalabrutinib 100mg Capsules (2022). Available online at: https://www.accessdata.fda.gov/drugsatfda_docs/label/2022/216387Orig2s000Correctedlbl.pdf (Accessed January 5, 2026).

[35] Wang M Salek D Belada D Song Y Jurczak W Kahl BS . Acalabrutinib Plus Bendamustine-Rituximab in Untreated Mantle Cell Lymphoma. J Clin Oncol. (2025) 43(20):2276–2284. doi: 10.1200/JCO-25-00690 PMC12225732 40311141

[36] Hallek M Cheson BD Catovsky D Caligaris-Cappio F Dighiero G Döhner H . iwCLL guidelines for diagnosis, indications for treatment, response assessment, and supportive management of CLL. Blood. (2018) 131:2745–60. doi: 10.1182/blood-2017-09-806398 29540348

[37] Cheson BD Fisher RI Barrington SF Cavalli F Schwartz LH Zucca E . Recommendations for initial evaluation, staging, and response assessment of Hodgkin and non-Hodgkin lymphoma: The Lugano classification. J Clin Oncol. (2014) 32:3059–67. doi: 10.1200/jco.2013.54.8800 25113753 PMC4979083

[38] group IC-Iw . An international prognostic index for patients with chronic lymphocytic leukaemia (CLL-IPI): A meta-analysis of individual patient data. Lancet Oncol. (2016) 17:779–90. doi: 10.1016/S1470-2045(16)30029-8 27185642

[39] Geisler CH Kolstad A Laurell A Räty R Jerkeman M Eriksson M . The Mantle Cell Lymphoma International Prognostic Index (MIPI) is superior to the International Prognostic Index (IPI) in predicting survival following intensive first-line immunochemotherapy and autologous stem cell transplantation (ASCT). Blood. (2010) 115:1530–9. doi: 10.1182/blood-2009-08-236570 20032504

[40] Sharman JP Egyed M Jurczak W Skarbnik A Pagel JM Flinn IW . Acalabrutinib with or without obinutuzumab versus chlorambucil and obinutuzumab for treatment-naive chronic lymphocytic leukaemia (ELEVATE-TN): A randomised, controlled, phase 3 trial. Lancet. (2020) 395:1278–91. doi: 10.1016/s0140-6736(20)30262-2 32305093 PMC8151619

[41] Yazdy MS Mato AR Roeker LE Jarral U Ujjani CS Shadman M . Toxicities and outcomes of acalabrutinib-treated patients with chronic lymphocytic leukemia: A retrospective analysis of real world patients. Blood. (2019) 134:4311. doi: 10.1182/blood-2019-130062

[42] Patel K Teschemaker A Tian H Pyrih N Kolvenbag G Patel S . Real-world effectiveness and safety outcomes of acalabrutinib treatment in patients with relapsed/refractory mantle cell lymphoma. Blood. (2024) 144:7744. doi: 10.1182/blood-2024-194523

[43] Andrew Lipsky NL . Managing toxicities of Bruton tyrosine kinase inhibitors. Hematol Am Soc Hematol Educ Program. (2020) 1:336–45. doi: 10.1182/hematology.2020000118 33275698 PMC7727553

[44] Ozturk E Erdogan Ozunal I . A rare side effect of ibrutinib: tumor lysis syndrome. (2021) 36:176–9. 10.5222/MMJ.2021.56424 PMC8226401 34239769

[45] Abbas HA Wierda WG . Acalabrutinib: A selective Bruton tyrosine kinase inhibitor for the treatment of B-cell Malignancies. Front Oncol. (2021) 11:668162. doi: 10.3389/fonc.2021.668162 34055635 PMC8162209

[46] Sharman JP Egyed M Jurczak W Skarbnik A Pagel JM Flinn IW . Efficacy and safety in a 4-year follow-up of the ELEVATE-TN study comparing acalabrutinib with or without obinutuzumab versus obinutuzumab plus chlorambucil in treatment-naïve chronic lymphocytic leukemia. Leukemia. (2022) 36:1171–5. doi: 10.1038/s41375-021-01485-x 34974526 PMC8979808

[47] Park D Chan-Golston AM Akhtari M . Meta-analysis of the efficacy and adverse effects of acalabrutinib in the treatment of relapsed/refractory chronic lymphocytic leukemia. Blood. (2023) 142:6553. doi: 10.1182/blood-2023-191104

[48] Byrd JC Harrington B O’Brien S Jones JA Schuh A Devereux S . Acalabrutinib (ACP-196) in relapsed chronic lymphocytic leukemia. N Engl J Med. (2016) 374:323–32. doi: 10.1056/nejmoa1509981 26641137 PMC4862586

[49] Woyach JA Smucker K Smith LL Lozanski A Zhong Y Ruppert AS . Prolonged lymphocytosis during ibrutinib therapy is associated with distinct molecular characteristics and does not indicate a suboptimal response to therapy. Blood J Am Soc Hematol. (2014) 123:1810–7. doi: 10.1182/blood-2013-09-527853 24415539 PMC3962160

