# Supplementary material for: A prospective, multi-center, phase IV clinical study to assess the safety and clinical outcome of acalabrutinib in Indian adult patients with chronic lymphocytic leukemia/small lymphocytic lymphoma or relapsed/refractory mantle cell lymphoma
Source: Front Oncol. 2026 Sep 10;16:1822178. doi: 10.3389/fonc.2026.1822178 (PMC13600985; doi:10.3389/fonc.2026.1822178)
Supplement: Supplementary file 1 [file DataSheet1.docx]

Supplementary Material

# 1 Supplementary Data

## 1.1 Supplementary Text Box: Inclusion/Exclusion Criteria

For inclusion in the study, participants had to fulfill all the following criteria:

1. Men and Women aged 18 years or more.
2. Eastern Cooperative Oncology Group (ECOG) performance status of 0, 1, or 2.
3. Able to receive all outpatient treatments, all laboratory monitoring, and all radiologic evaluations.
4. The following laboratory parameters:
a) Absolute neutrophil count (ANC) ≥750 cells/μL or ≥500 cells/μL in patients with documented bone marrow involvement and independent of growth factor support 7 days before the assessment.
b) Platelet count ≥50,000 cells/μL or ≥30,000 cells/μL in patients with documented bone marrow involvement and without transfusion support 7 days before the assessment.
c) Aspartate transaminase (AST) and alanine transaminase (ALT) ≤2.0 x upper normal limits (ULN).
d) Total bilirubin ≤1.5 x ULN.
e) Estimated creatinine clearance of ≥30 mL/min.
5. Refractory disease, defined as achieving less than partial response with the most recent treatment within six months before study entry.
6. Provision of signed, written, and dated informed consent prior to any study-specific Procedures.
7. The patients of either CLL or MCL:
a. CLL patients:
i. Treatment naïve or one or more prior systemic therapy for CLL.
ii. Diagnosis of CD20+ CLL that met published diagnostic criteria [35]
iii. An active disease that met ≥1 of the following International Workshop on Chronic Lymphocytic Leukemia (iwCLL)2018 criteria for requiring treatment:
1) Evidence of progressive marrow failure as manifested by the development of, or worsening of, anemia or thrombocytopenia. Cut-off levels of Hb ˂10 g/dL or platelet counts ˂100 × 10^9^ /L are generally regarded as an indication for treatment. However, in some patients, platelet counts <100 × 10^9^/L may remain stable over a long period; this situation does not automatically require therapeutic intervention.
2) Massive (i.e., ≥6 cm below the left costal margin) or progressive or symptomatic splenomegaly.
3) Massive nodes (i.e., ≥10 cm in longest diameter) or progressive or symptomatic lymphadenopathy.
4) Progressive lymphocytosis with an increase of ≥50% over a 2-month period or Lymphocyte Doubling Time (LDT) in ˂6 months. LDT can be obtained by linear regression extrapolation of absolute lymphocyte counts obtained at intervals of 2 weeks over an observation period of 2 to 3 months; patients with initial blood lymphocyte counts ˂30 × 10^9^ /L may require a longer observation period to determine the LDT. Factors contributing to lymphocytosis other than CLL (e.g., infections, steroid administration) should be excluded.
5) Autoimmune complications, including anemia or thrombocytopenia, poorly responsive to corticosteroids.
6) Symptomatic or functional extra-nodal involvement (e.g., skin, kidney, lung, spine).
7) Disease-related symptoms as defined by any of the following:
a) Unintentional weight loss of ≥10% within the previous 6 months.
b) Significant fatigue (i.e., ECOG performance scale 2 or worse; cannot work or unable to perform usual activities).
c) Fever ≥100.5°F or 38.0°C for 2 or more weeks without evidence of infection.
d) Night sweats for ≥1 month without evidence of infection.
b. MCL Patients:
i. Confirmed MCL with translocation t(11;14) (q13;q32) and/or overexpressed cyclin D1
ii. Measurable nodal disease (one or more lesions measuring ≥2 cm in the longest diameter)
iii. Relapsed after, or were refractory to, 1-5 previous treatments.

Any of the following were regarded as a criterion for exclusion from the study:

1. Known prolymphocytic leukemia, central nervous system (CNS) lymphoma or leukemia; or known history of (or currently suspected) Richter’s syndrome
2. Treatment with chemotherapy, external beam radiation therapy, anticancer antibodies, or investigational drug within 30 days of the first dose of study drug
3. Prior radio-conjugated or toxin-conjugated antibody therapy
4. Anticoagulation therapy (e.g., warfarin or equivalent vitamin K antagonists) within 7 days of the first dose of the study drug.
5. Major surgery ≤30 days before the first dose of study drug
6. History of stroke or intracranial hemorrhage ≤6 months before the first dose of the study drug
7. History of bleeding diathesis
8. Prior exposure to a B-cell lymphoma-2 (Bcl-2) inhibitor or B-cell receptor inhibitor like BTKs
9. Active cytomegalovirus (CMV) infection or serologic status reflecting active Hepatitis B or C infection or known history of infection with human Immunodeficiency virus (HIV) or any uncontrolled active systemic infection.
10. Significant cardiovascular disease such as uncontrolled or symptomatic arrhythmias, congestive heart failure, or myocardial infarction within 06 months of screening, or any Class 3 or 4 cardiac diseases as defined by the New York Heart Association Functional Classification, or QTcB >480 msec at screening.
11. Requiring treatment with proton-pump inhibitors (e.g., omeprazole, esomeprazole, lansoprazole, dexlansoprazole, rabeprazole, or pantoprazole).
12. Breastfeeding or pregnant.
13. Life-threatening illness, medical condition, or organ/system dysfunction which, in the Investigator’s opinion, could have compromised the subject’s safety or put the study at risk.
14. Concurrent participation in another therapeutic clinical study.

# 2 Supplementary Figures and Tables

## 2.1 Supplementary Figures

Abbreviations: EOT, End of treatment; BID, Twice a day.

Annotations:

- Patients were to sign the informed consent form (ICF) before any study-specific procedures were performed.
- Procedures conducted as part of the patient’s routine clinical management (e.g., blood count) and obtained before signing the ICF were utilized for screening or baseline purposes provided they met the protocol-specified criteria and were performed within the time frame (-6 to 0 days).
- Treatment Phase from Day 1 to Day 168. The treatment with acalabrutinib was continued until disease progression or unacceptable drug-related toxicity, whichever occurs earlier.

**Supplementary Figure 1. Study Design**

## 2.2 Supplementary Table

## Supplementary Table 1. Health-related quality of life on EORTC QLQ-C30 questionnaire (FAS)

| **Total Score** | **Baseline (Visit 1)** | | | **End of treatment (Visit 8)** | | |
| --- | --- | --- | --- | --- | --- | --- |
|  | **CLL/SLL**  **(N=89)**  **n (%)** | **MCL**  **(N=11)**  **n (%)** | **Total**  **(N=100)**  **n (%)** | **CLL/SLL**  **(N=89)**  **n (%)** | **MCL**  **(N=11)**  **n (%)** | **Total**  **(N=100)**  **n (%)** |
| n | 89 | 11 | 100 | 83 | 8 | 91 |
| Missing | 0 | 0 | 0 | 0 | 0 | 0 |
| Mean (SD) | 56.9 (12.52) | 57.0 (6.46) | 56.9 (12.00) | 57.9 (9.34) | 58.6 (6.76) | 58.0 (9.13) |
| Median | 61.3 | 61.5 | 61.3 | 61.3 | 61.7 | 61.3 |
| (min, max) | (5.50, 88.00) | (43.00, 62.10) | (5.50, 88.00) | (12.00, 93.00) | (42.00, 62.07) | (12.00, 93.00) |

Annotations:

- ‘Health-related quality of life’ was assessed using the EORTC QLQ-C30 questionnaire at the screening visit (Visit 1), during the treatment (Visit 5), and at the end of the study (visit 8)
- “N” in the column header represents the total number in the FAS.
- "n” in summary statistics represents the total number of patients.

Abbreviations: CLL, chronic lymphocytic leukemia; SLL, small lymphocytic lymphoma; FAS, full analysis set.
